# Extraordinary Multi-Organismal Interactions Involving Bacteriophages, Bacteria, Fungi, and Rotifers: Quadruple Microbial Trophic Network in Water Droplets

**DOI:** 10.3390/ijms22042178

**Published:** 2021-02-22

**Authors:** Katarzyna Turnau, Edyta Fiałkowska, Rafał Ważny, Piotr Rozpądek, Grzegorz Tylko, Sylwia Bloch, Bożena Nejman-Faleńczyk, Michał Grabski, Alicja Węgrzyn, Grzegorz Węgrzyn

**Affiliations:** 1 Institute of Environmental Sciences, Jagiellonian University in Krakow, Gronostajowa 7, 30-387 Krakow, Poland; edyta.fijalkowska@uj.edu.pl; 2 Malopolska Centre of Biotechnology, Jagiellonian University in Krakow, Gronostajowa 7a, 30-387 Krakow, Poland; rafal.wazny@uj.edu.pl (R.W.); piotr.rozpadek@uj.edu.pl (P.R.); 3 Institute of Zoology and Biomedical Research, Jagiellonian University in Krakow, Gronostajowa 7, 30-387 Krakow, Poland; grzegorz.tylko@uj.edu.pl; 4 Laboratory of Phage Therapy, Institute of Biochemistry and Biophysics, Polish Academy of Sciences, Kladki 24, 80-822 Gdansk, Poland; sylwia.bloch@ug.edu.pl (S.B.); alicja.wegrzyn@ug.edu.pl (A.W.); 5 Department of Molecular Biology, Faculty of Biology, University of Gdansk, Wita Stwosza 59, 80-308 Gdansk, Poland; bozena.nejman-falenczyk@ug.edu.pl (B.N.-F.); michal.grabski@phdstud.ug.edu.pl (M.G.); grzegorz.wegrzyn@biol.ug.edu.pl (G.W.)

**Keywords:** rotifers, predatory fungi, bacteria, bacteriophages, extraordinary trophic network

## Abstract

Our observations of predatory fungi trapping rotifers in activated sludge and laboratory culture allowed us to discover a complicated trophic network that includes predatory fungi armed with bacteria and bacteriophages and the rotifers they prey on. Such a network seems to be common in various habitats, although it remains mostly unknown due to its microscopic size. In this study, we isolated and identified fungi and bacteria from activated sludge. We also noticed abundant, virus-like particles in the environment. The fungus developed absorptive hyphae within the prey. The bacteria showed the ability to enter and exit from the hyphae (e.g., from the traps into the caught prey). Our observations indicate that the bacteria and the fungus share nutrients obtained from the rotifer. To narrow the range of bacterial strains isolated from the mycelium, the effects of bacteria supernatants and lysed bacteria were studied. Bacteria isolated from the fungus were capable of immobilizing the rotifer. The strongest negative effect on rotifer mobility was shown by a mixture of *Bacillus* sp. and *Stenotrophomonas maltophilia*. The involvement of bacteriophages in rotifer hunting was demonstrated based on molecular analyses and was discussed. The described case seems to be an extraordinary quadruple microbiological puzzle that has not been described and is still far from being understood.

## 1. Introduction

Interactions between more or less phylogenetically distant organisms evolved from the beginning of life on Earth and jointly coevolved toward the establishment of a functional unity called a symbiom [1]. In various forms, undescribed symbiosis may be encountered, especially at sites best hidden from the naked eye. Activated sludge is an extraordinary microenvironment with a rich biodiversity of organisms involved in complex interplays. Investigating interactions between members of this complicated community can help us understand the processes that drive evolution in the microscopic world. Such microbial entities may include diverse creatures such as rotifers, protists, fungi, bacteria, and bacteriophages.

Rotifers such as *Lecane inermis* are indicators of well-functioning sediment and contribute to better flocculation of sludge by limiting the density of filamentous bacteria, which, when uncontrolled, cause sludge bulking. Rotifers nourish on non-flocculated filamentous bacteria and suspended particles of organic matter that are inedible for almost all protozoa and other invertebrates. This makes rotifers essential components of activated sludge [2]. In nature, however, every organism has its enemies.

Different organisms utilize diverse strategies to obtain necessary nutrients ranging from the decomposition of organic matter (saprotrophy), pathogenicity, mutualism, or predation. In many cases (e.g., upon environmental cues), the trophic behavior of a particular organism can change. While fungi and bacteria are grazed by many animals including protozoa, mites, insects, nematodes, etc., they can either evolve strategies to secure themselves or attack their enemies. Predatory bacteria attack other bacteria or fungi; fungi can obtain nutrients from bacteria by lysis and from other organisms such as rotifers. Predation in bacteria and fungi has evolved many times and in both kingdoms independently [3,4,5].

Predatory fungi of the subdivision Zoopagomycota [6], which inhabit activated sludge, have become specialized in rotifer hunting. Around 300 taxa of predatory fungi that belong to the Zoopagomycota, Ascomycota (including anamorph fungi), and Basidiomycota have been described so far. Our knowledge of predatory Zoopagomycota fungi is very limited, both in taxonomy and ecology [7]. In recent years, only fungal predators of nematodes have received significant interest due to their potential utilization in the biocontrol of nematodes in agricultural soils.

To catch its victim, the fungus needs to develop effective traps. The phenotypical diversity of the trapping organs is so large that it is often used as a taxonomic criterion for the identification of these fungi. The best-known case is Orbilliaceae (Ascomycota), and the differences in the phenotype are supported by molecular phylogenetic analysis of nucleotide sequences of three protein-coding genes and ribosomal DNA [8]. Trapping devices of predatory fungi evolved into two parallel lineages characterized by two distinct trapping mechanisms: (1) non-adhesive and (2) adhesive. These structures most likely evolved in saprobic fungi due to decaying wood that is always limited in available nitrogen [9]. By developing a new strategy, fungi have acquired a competitive advantage by direct access to nutrients from the entrapped prey [10,11,12].

Among the predatory fungi, Zoopagomycota are the least described. Their relationship (as well as other fungal predators) with other potentially symbiotic microorganisms such as bacteria has not been thoroughly investigated. According to available reports, bacteria residing in proximity to or on the surface of these fungi produce urea, which stimulates the development of traps that catch the nematode [13]. 

Endophytic bacteria are common in the Mucoromycotina and Zoopagomycotina [14]. There are known phenotypes characteristic of the presence of bacterial endosymbionts in rhizomes. A close relative of *Zoophagus insidians*, *Rhizopus microsporus*, which is most often associated with saprobic activity on bread, thanks to endosymbiotic bacteria, acquires the ability to produce a toxin called rhizoxin that may affect plant growth and transition the fungus to a pathogenic mode [15,16,17,18].

Interactions between bacteria and eukaryotic organisms in the environment include the participation of bacteriophages. One of the most well-studied examples is *Escherichia coli* strains bearing Shiga toxin-converting bacteriophages, as described and discussed previously [19,20]. *E. coli* is used as prey by various eukaryotic organisms including some nematodes (like *Caenorhabditis elegans*) and protists (like *Tetrahymena* sp.), which graze on them. To capture bacteria, protists often use a sophisticated strategy to excrete hydrogen peroxide, which is deleterious to the vast majority of prokaryotic cells that, contrary to eukaryotes, lack catalases and enzymes that convert H_2_O_2_ to water and oxygen. This strategy is very effective in most cases; however, when *E. coli* lysogenize for Shiga, the toxin-converting bacteriophage is attacked, and prophage induction occurs in a small fraction of cells (efficiency of Shiga toxin-converting prophage induction by hydrogen peroxide is as low as 1%), which is sufficient to produce large amounts of the toxin. Following lysis of bacterial cells due to the lytic development of the bacteriophage, the toxin is released and kills the predator, which allows the majority of bacterial cells to survive; therefore, a small fraction of the bacterial population is sacrificed when producing Shiga toxin after prophage induction, but this is beneficial for the whole population since the rest of the bacterial cells can escape the attack of the predator, which is killed by the toxin. This phenomenon has been called bacterial altruism [20], and is considered an important part of the sociobiology of bacteriophages, bacteria, and eukaryotic organisms [21].

In this study, we isolated and identified the predatory fungus *Zoophagus insidians*, appearing in activated sludge from a wastewater treatment plant. We attempted to unveil the morphological, ecological, and physiological interactions between the fungus and other associated microbes. This includes bacteriophages, whose involvement could be demonstrated based on molecular studies.

## 2. Results

### 2.1. Isolation and Molecular Identification of the Fungus and Morphological Description of the Microbial Community

**Figure 1:**
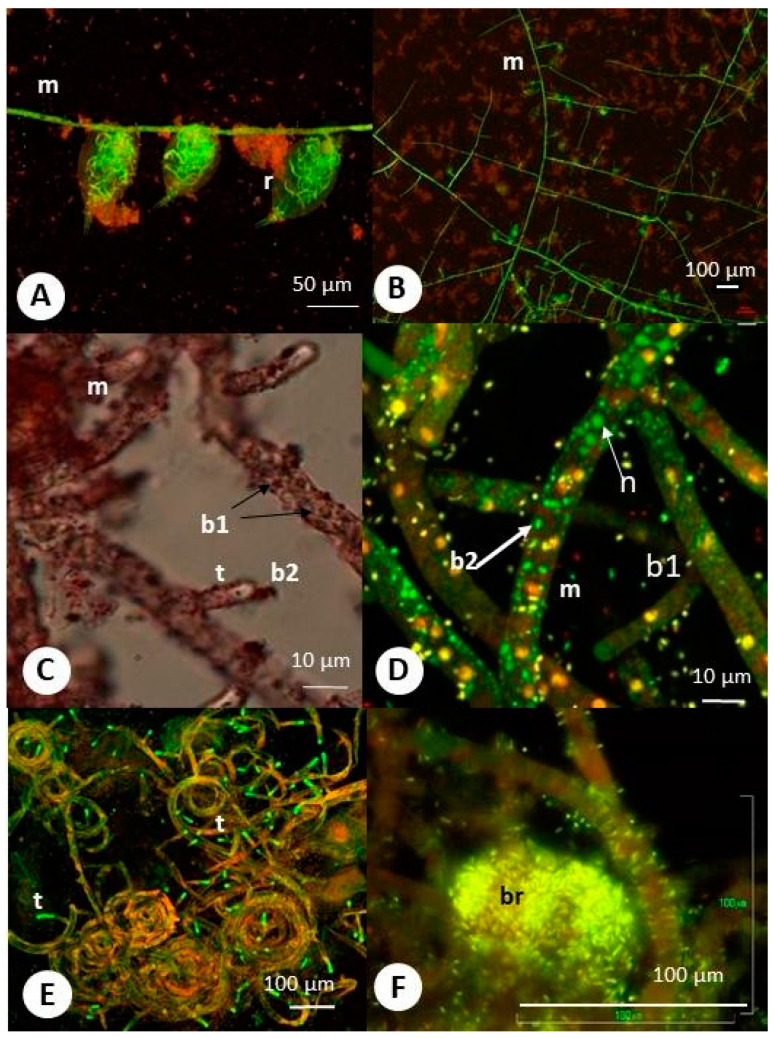
Predatory fungus (*Zoophagus insidians*), rotifers (*Lecane inermis*), and bacteria cultured in “Żywiec” water. (**A**) Rotifers trapped by fungus with absorptive hyphae developed within rotifer; (**B**) mycelium forming network on the surface of the glass; (**C**) mycelium with lipids stained with oil red O; (**D**) mycelium with traps and abundant bacteria; (**E**) coiled mycelia with numerous traps (green); (**F**) dead rotifer with bacteria inside; (**A**,**B**) and (**D**,**E**,**F**) material stained with Live–Dead Bacterial Kit; m—mycelium; c—mycelial coil; t—green stained traps; n—nucleus; b1—bacteria inside mycelium; b2—bacteria outside mycelium; br—bacteria within rotifer; r—rotifer; t—trap.

The predatory fungus was isolated from samples of activated sludge from nitrification chambers of a wastewater treatment plant located in the south of Poland in 2015. According to morphological features, the fungus was classified as *Zoophagus* sp. [22], where the main criterion distinguishing *Zoophagus* from *Lecophagus* was the septation of mycelium and conidia. According to the sequence of the 18s rDNA region, the fungus was identified as *Zoophagus insidians*. In culture, the fungal mycelium branched from the bottom of the dish and formed conspicuous coils, protruding into the medium. The mycelium formed an organized network of spirally entangled nodes connected with straight hyphae (Figure 1A–C). Club-shaped traps developed along the mycelium at irregular intervals. The traps were uniform in shape and not separated (at this stage) from the rest of mycelium, allowing a free flow of cytoplasm; however, empty traps (after protoplast retrieved back) were cut off by a rounded septum. On the surface of the mycelium, we noticed extremely abundant rod-like structures resembling bacteria. This was confirmed by staining with the LIVE/DEAD Bacterial Viability Kit; however, bacteria were visible outside the mycelium and inside the hyphae (Figure 1D). The bacteria differed from nuclei by size (nuclei were round and two times bigger). 

**Figure 2:**
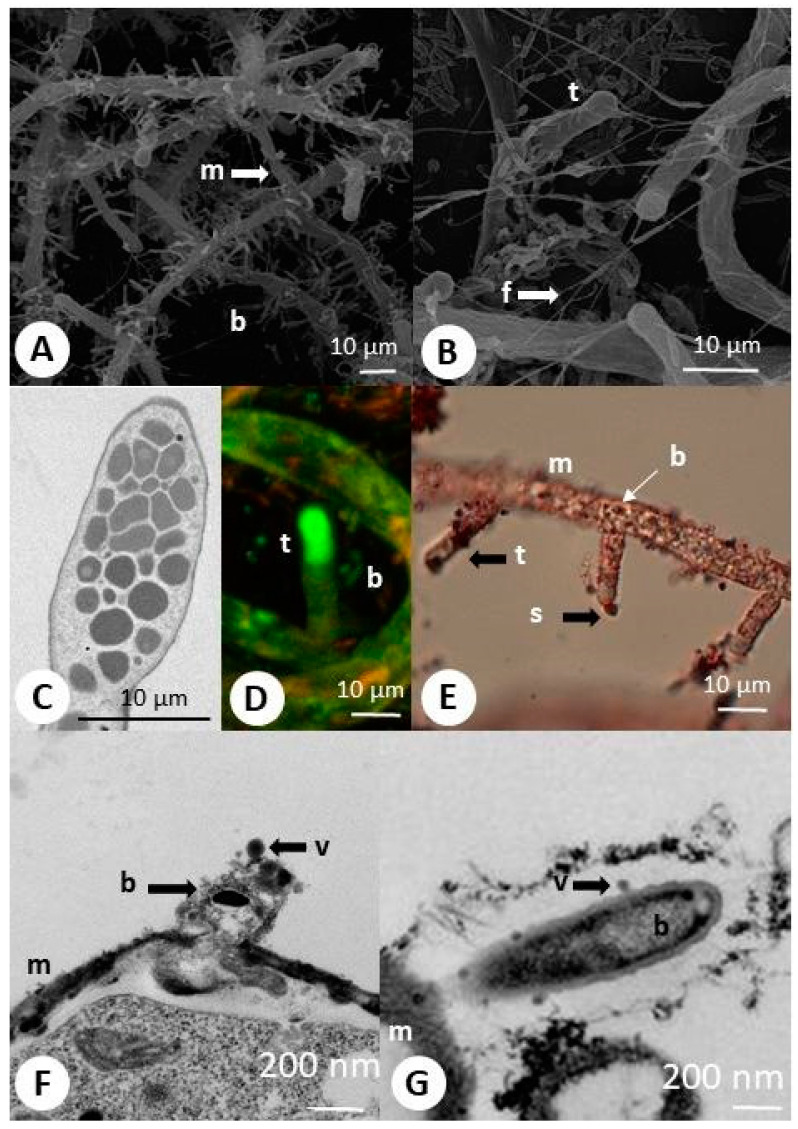
Predatory fungus (*Zoophagus insidians*), and bacteria cultured in “Żywiec” water. (**A**) Bacteria abundantly attached to fungal mycelium; (**B**) traps and mycelium with bacteria forming flagella-like structures; (**C**–**E**) mycelium with bottle-shaped traps: (**C**) TEM (transmission electron microscopy) micrograph showing the trap with numerous electron-dense bodies; (**D**) similar region seen stained with Live–Dead Bacterial Kit (confocal microscope); (**E**) mycelium with traps stained with oil red O (bacterial wall lipids stain red); (**F**,**G**) bacteria entering or leaving fungus with virus-like particles released from the bacterium; m—mycelium; t—trap; b—bacteria; v—virus-like particles; s—substance released from the trap; f—flagella-like structures.

**Figure 3:**
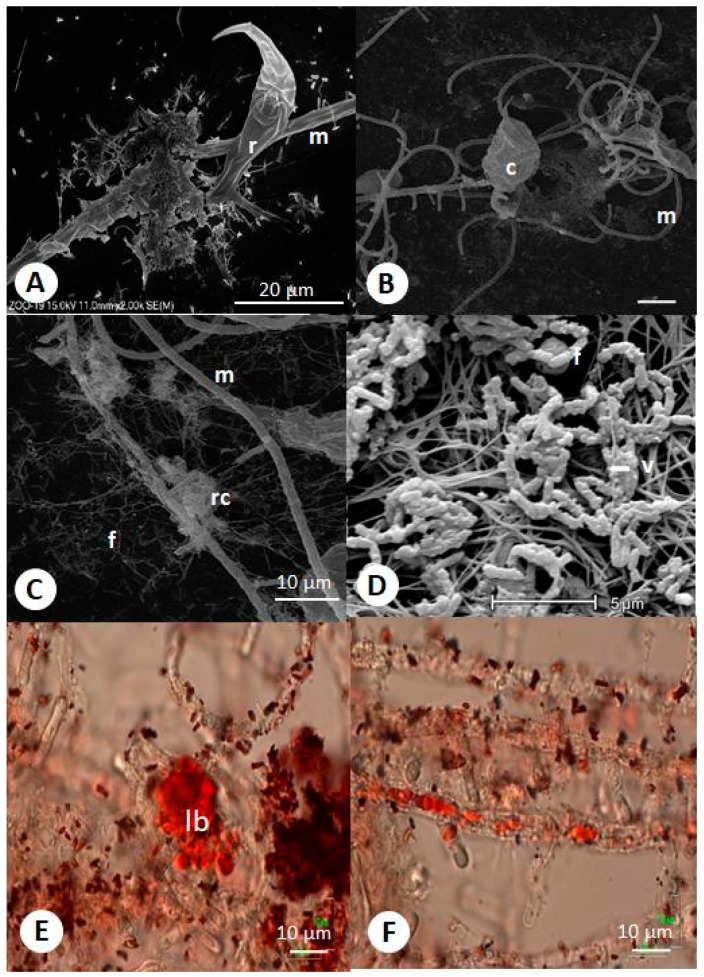
SEM (scanning electron microscopy) and LM (light microscopy) micrographs of *Zoophagus insidians* (predatory fungus), rotifers (*Lecane inermis*), and bacteria cultured in “Żywiec” water. (**A**) Rotifer attached to glue formed fungal and bacterial material and fungus on the glass surface; (**B**) rotifer trapped by fungus; (**C**) remnants of rotifers after digestion by fungi and bacteria; (**D**) bacteria with virus-like particles; (**E**) lipid bodies in rotifer stained with oil red O; (**F**) lipids transported from rotifer to mycelium; r—rotifer; c—immobilized rotifer; rc—remnants of cadaver and absorptive hyphae; m—mycelium; f—flagella-like structures; v—virus-like particles; Ib—lipid bodies.

Furthermore, contrary to nuclei, the bacteria were stained with oil red O (Figure 1C and Figure 2E). The vast majority of these bacteria were alive (green stained). The tips of the traps were uniformly green (Figure 1E and Figure 2D). Often, the bacteria were visible at the top of the trap (e.g., Figure 2E). Visualization with SEM (scanning electron microscopy) yielded a more precise view of the bacterial arrangement (Figure 2 and Figure 3). Most of them were attached to the hyphae (Figure 2A). The bacteria were often connected to the mycelium by fibrillary structures of up to 10 µm long (Figure 2B). TEM (transmission electron microscopy) observations confirmed the entrance/exit of bacteria to/from the mycelium (Figure 2C). The bacteria did not affect hyphae growth. Within the mycelium, besides the nuclei, mitochondria, paramural vesicles, and vacuoles, electron-dense bodies of up to 3 µm diameter were visible (Figure 2D). In addition, there were numerous much smaller electron-dense particles (ca. 0.5 µm). On several occasions, 50 nm virus-like particles resembling bacteriophages were observed on the bacteria’s surface (Figure 2E,F). Such particles were also found with SEM on the surface of degraded bacteria (Figure 3) attached to the hyphae. 

### 2.2. “Headhunting” in Rotifer Culture

After approaching the fungus, rotifers attached the hyphae by catching the tip of a trap. From this moment, the rotifers were entrapped by the fungus. According to observations, the mycelium grew inside the immobilized rotifer. Bacteria were visible inside the hyphae developing within the rotifer during the initial stages of fungal invasion and later migrating into the rotifer (Figure 1F). It seemed that the carcasses became filled with bacteria that possibly spread inside the rotifer from inside the mycelium. The absorptive hyphae took up lipids from the rotifers and transferred them (lipids were visible first in rotifers and then within hyphae in high abundance after catching the rotifer by staining with oil O) into the mycelium (Figure 3E,F). Subsequently, the fungus retrieved its cytoplasm back out of the rotifer and formed a cross wall at the bottom of the trap. Up to 50 rotifers were seen to become immobilized at sites where the fungus formed clusters of traps on a spirally-shaped mycelium.

### 2.3. Identification of Bacteria Associated with Zoophagus insidians 

To identify culturable bacteria abundantly present inside the fungus, three different modes of endohyphal bacteria isolation were utilized: (1) direct amplification DNA from water-sterilized mycelium; (2) amplification of DNA of pure bacterial cultures isolated from water-sterilized mycelium; and (3) amplification of DNA of pure bacterial cultures isolated from antibiotic-sterilized mycelium.

Bacterial strains were identified according to their 16S rDNA sequence and, if necessary, with the rpoB and/or 16S-23S DNA sequence (Table 1). 

### 2.4. Co-Culture of Rotifers with Zoophagus Symbiotic Bacteria and Bacterial Lysates Affected Rotifer Mobility Differently

**Figure 4:**
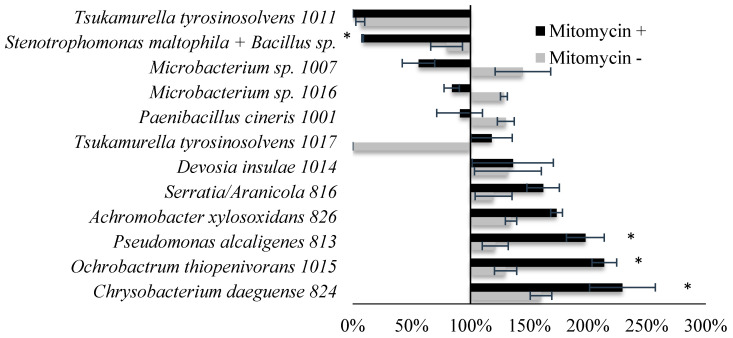
Mobility index of rotifers treated with phage product of bacterial culture (mitomycin+) and bacterial culture (mitomycin−) calculated in comparison to the control (treated only with TSB (tryptic soy broth) medium; asterisks (*) indicate statistically significant differences between mitomycin− and mitomycin+ treatments based on a *t*-test (*p* ≤ 0.05).

In order to verify whether bacteria associated with *Zoophagus* may be involved in hunting for rotifers, rotifer cultures were supplemented with a bacterial culture supernatant (Figure 4). Within the next few hours after the treatment, the number of immobilized rotifers was assessed. The supernatant from the following bacterial cultures accelerated rotifer movement: *Pseudomonas alcaligenes*, *Ochrobactrum thiopenivorans*, and *Chrysobacterium daeguense*. Three bacterial strains of *Tsukamurella tyrosinosolvens* and a mixture of strains containing *Stenotrophomonas maltophilia* and *Bacillus* sp. closely related to *B. siralis* had a significant negative effect on rotifer mobility, causing almost immediate immobilization of the cultured rotifers. *T. tyrosinosolvens* and *B. siralis* were of a similar bacilloid shape and both unknown as to their ability to produce toxins. Supernatant from the remaining cultures did not alter rotifer mobility (Figure 4). Since various bacteriophages carry genes coding for proteins deleterious for various eukaryotic organisms, from protists to mammals [20,23], we hypothesized that bacteria associated with *Zoophagus* might carry prophages bearing such genes. If so, after prophage induction, these genes might be expressed, causing the production of proteins either inhibiting rotifer mobility or influencing other biological processes of these animals. To test if isolates of bacteria are lysogenic for bacteriophages, we provoked the phage lytic development by adding mitomycin C to the bacterial cultures. Interestingly, in some cases, we observed the lysis of bacterial cells, suggesting that the tested bacteria contained phage DNA integrated into the chromosome (Table S1). Thus, to verify whether bacteriophages abundantly present in *Zoophagus* cultures (Figure 4) participated in hunting for rotifers, rotifer cultures were supplemented with a bacterial suspension treated with mitomycin C. Mitomycin C treatment was used to induce putative prophage from bacteria isolated from *Zoophagus*. Application of both strains of *T. tyrosinosolvens* had a rapid immobilizing effect on rotifers; however, lysates of different *T. tyrosinosolvens* strains induced different effects on rotifer mobility. Lysate from strain 1011 inhibited rotifer mobility, whereas lysate from strain 1017 did not affect the mobility of the rotifers. In the case of the mixture of *Bacillus* sp. and *S. maltophilia*, the effect of the lysate was significantly stronger in comparison to the effect of culture supernatant, and that supported the involvement of a virus in rotifer intoxication (Figure 4). 

### 2.5. Newly Identified Bacillus sp. Phage

Taking into account that the application of the supernatant of *Bacillus* sp. 1020A and *S. maltophilia* 1020B, obtained after treatment of the bacterial mixture with mitomycin C, affected the mobility of rotifers more than the culture supernatant without mitomycin C, we decided to test if these two isolates of bacteria were lysogenic for bacteriophages or not. As mitomycin C is a known and widely-used prophage-inducing factor, we provoked the putative phage lytic development by adding an antibiotic to the bacterial cultures. In this way, we analyzed bacterial strains for lysis capacity. Interestingly, the lysis of bacterial cells occurred only in the *Bacillus* sp. 1020A culture, which may indicate the presence of phage DNA integrated into the bacterial chromosome. 

**Figure 5:**
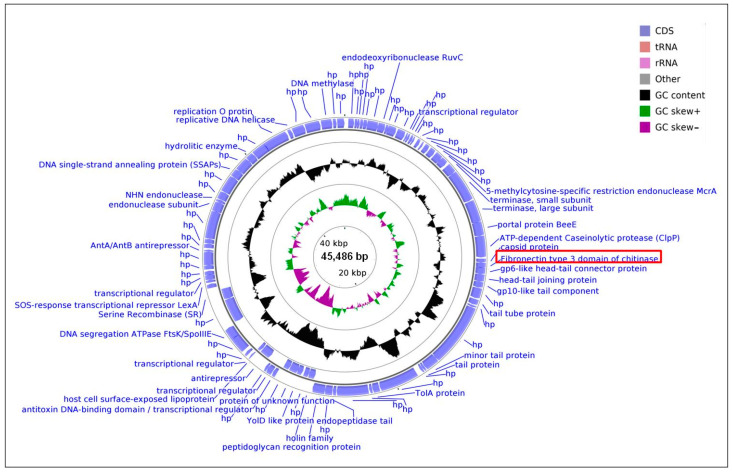
Genome structure of *Bacillus* bacteriophage created by using the BRIG platform and the CGView program. The outermost ring presents open reading frames (ORFs) with predicted annotations (blue arrows). The middle ring shows the GC content (black). The innermost ring represented GC skew+ (green) and GC skew− (purple). The ORF26 that potentially encodes the protein containing fibronectin type 3 domain, characteristic for/to chitinase, is indicated by the red frame.

The whole genome of the temperate vB_Bacillus_1020A virus was sequenced and deposited in GenBank (the NCBI (National Center for Biotechnology Information) accession number is MT210152). Interestingly, by using the vB_Bacillus_1020A phage genome as queries to search against the BLASTN (Basic Local Alignment Search Tool Nucleotide) database, we did not find any homologous contig and genome, further indicating the novelty of this vB_Bacillus_1020A bacteriophage (Figure 5). Moreover, we also tested the host range of phage vB_Bacillus_1020A. Unfortunately, this temperate virus did not infect any of the tested *Bacillus* strains: *B. cereus*, *B. megaterium*, *B. firmus*, *B. siralis*, and *B. subtilis*; therefore, we concluded that this vB_Bacillus_1020A phage is probably a narrow host range virus.

Bioinformatic analyses revealed that vB_Bacillus_1020A has a double-stranded DNA, 45,486 in length, with an overall G + C content of 37.5%. No tRNA-encoding genes were detected. Among all 85 identified putative open reading frames (ORFs), 76 ORFs were located on the direct strand of the phage genome, and nine ORFs were on the complementary strand (Figure 5). About 44.7% of the ORFs (38 ORFs) were assigned a putative function based on their amino acid sequence homology to known proteins or evolutionarily conserved protein domains and motifs. Homologous of the remaining 47 ORFs (approximately 55.3%) were uncharacterized proteins. Proteins encoded by these 38 ORFs include structural proteins (phage capsid protein, head-tail connector proteins, putative tail component, tail tube protein, minor tail protein, tail protein, and endopeptidase) and proteins involved in processes such as DNA replication (replicative DNA helicase and replication O protein), DNA packaging (small and large subunits of terminase), and cell lysis (hydrolytic enzyme and holin). The genome of vB_Bacillus_1020A also contained sequences encoding the main markers of temperate viruses like prophage antirepressor (ORF52), serine recombinase (ORF59), or AntA/AntB antirepressor (ORF66). 

Moreover, the results obtained after the genetic screening of the vB_Bacillus_1020A genome against the Virulence Factors of Pathogenic Bacteria database suggest that this virus is free of antimicrobial-resistant genes and the virulent factors homolog. Interestingly, the analysis of the vB_Bacillus_1020A genome showed that ORF26 probably encodes the protein containing the fibronectin type 3 domain of chitinase. This suggested that bacteriophage vB_Bacillus_1020A might influence the rotifers’ biology by expressing a gene coding for chitinase, which, after its release from the bacterial host cells, can affect these animals.

### 2.6. Chitinase Activity of Isolated Strains of Bacteria

Bacterial strains isolated from *Z. insidians* were verified for chitinase activity using agar media containing colloid chitin (Table 2). Bacterial strains not showing chitinase activity accounted for 28% (including *Bacillus* sp.), with low and high activity of 31% and 41%, respectively.

### 2.7. Chitinase Activity of Isolated Strains of Bacillus sp.

Although *Bacillus* sp. did not show chitinase activity in the agar test, the genomic study of its bacteriophage showed the presence of ORF26, which potentially encodes the protein containing the fibronectin type 3 domain, characteristic for/to chitinase. To confirm that *Bacillus* sp. produces chitinase, we verified its chitinolytic activity using the Fluorimetric Chitinase Assay Kit (Sigma-Aldrich, St. Louis, MO, USA). This assay provides three different substrates for the detection of the exochitinase (4-Methylumbelliferyl N-acetyl-β-D-glucosaminide and 4-Methylumbelliferyl N, N’-diacetylchitobioside hydrate) and endochitinase (4-Methylumbelliferyl β-D-N, N’, N’’-triacetylchitotriose) activity.

**Figure 6:**
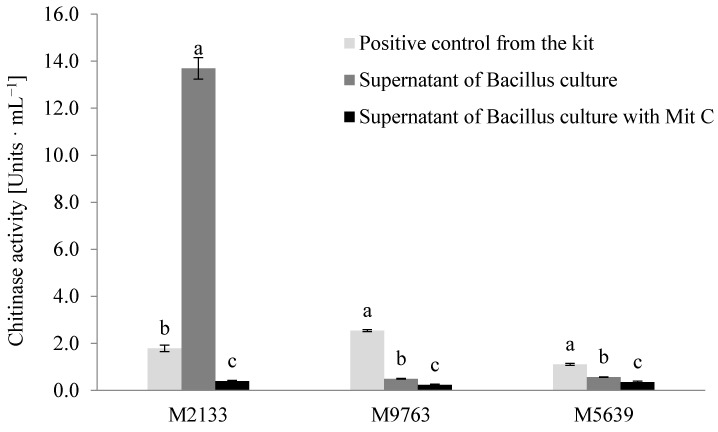
The activity of the chitinase produced by *Bacillus* sp.; positive control provided with the kit is represented by a light grey bar; the results obtained for the bacterial samples without an induction agent are marked as a dark grey bar; the activity of the enzyme in bacterial samples, in which the induction of prophage was provoked by mitomycin C, are marked as a black bar; names: M2133 and M9763 indicate substrates used for detection of exochitinase activities (β-N-acetylglucosaminidase and chitobiosidase, respectively) whereas M5639 indicate the substrate used for endochitinase activity; the presented results are mean values from three independent experiments. Error bars indicate SD (standard deviation) and statistical significance was tested by the one-way analysis of variance (ANOVA) followed by the Tuckey *post-hoc* test at *p* ≤ 0.05; bars topped by the same letter (a, b or c) do not differ significantly.

We observed that the highest activity of the enzyme was detected in the culture containing the 4-Methylumbelliferyl N-acetyl-β-d-glucosaminide substrate and the supernatant obtained after centrifugation of *Bacillus* bacterial cells. Moreover, we noticed that the chitinase produced by *Bacillus* sp. is not specific for 4-Methylumbelliferyl N, N’-diacetylchitobioside hydrate and 4-Methylumbelliferyl β-D-N, N’, N’’-triacetylchitotriose (Figure 6).

### 2.8. Rotifer Reaction to Chitinase

To verify whether the rotifers reacted to the enzyme, we treated rotifer cultures with commercially available bacterial chitinase. The chitinase effect was not obvious as even the introduction of the buffer resulted in immobilization of the rotifers; however, when the cultures were inspected later, those treated with chitinase were strongly changed compared to the control and buffer treated samples. We still do not know if the enzyme concentration was representative of the rotifer–bacteria model investigated here. In the next step, we evaluated the chitinase activity of obtained bacterial isolates on agar plates supplemented with chitin. Several strains showed strong activity of this enzyme. The strongest was shown by *S. maltophilia* (Table 2).

**Figure 7:**
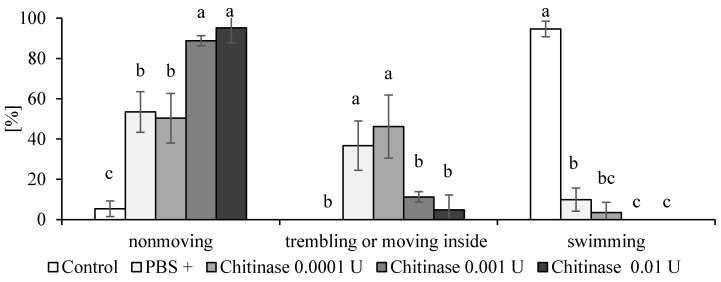
The reaction of the rotifers two hours after the introduction of different concentrations of chitinase; control—no treatment; PBS (phosphate buffered saline) only; the presented results are mean values from five replicates with error bars indicating SD, statistical significance was tested by the one-way analysis of variance (ANOVA) followed by the Tuckey post-hoc test at *p* ≤ 0.05; bars topped by the same letter (a, b or c) do not differ significantly.

**Figure 8:**
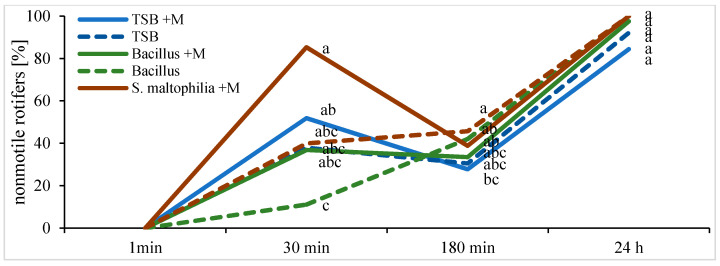
Effect of tryptic soy broth (TSB), bacterial (*Bacillus* and *S. maltophilia*) supernatant, and bacterial lysates obtained after treatment of bacterial cultures with mitomycin C (+M), on rotifer motility; observations performed up to 24 h. Statistical significance was tested by the one-way analysis of variance (ANOVA) followed by the Tuckey post-hoc test at *p* ≤ 0.05 (N = 5); points topped by the same letter (a, b or c) do not differ significantly.

**Figure 9:**
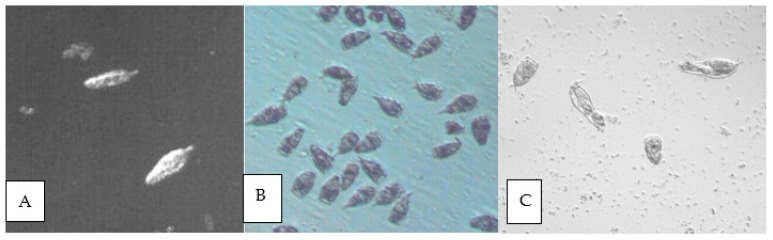
Rotifers treated with chitinase: (**A**) swimming rotifers before treatment; (**B**) immobilized rotifers with mastax inside, directly after treatment; (**C**) after 24 h; few remain alive and most strongly changed, without visible internal structures, but still swimming; magnification 100×.

There were very few differences between the effects of bacterial supernatants (mitomycin−), bacterial lysates obtained after treatment of bacterial culture with mitomycin C, and the percentage of viable but nonmotile rotifers; however, at the highest concentration of chitinase, differences in the morphology of swimming rotifers in comparison to other samples were visible (Figure 7 and Figure 8). All the treatments including TSB (tryptic soy broth) medium, resulted in pulling the mastax (Figure 9) inside the rotifer, which was similar in effect to the rotifers trapped by the fungus.

### 2.9. Cyanogenic Activity of Selected Strains

**Figure 10:**
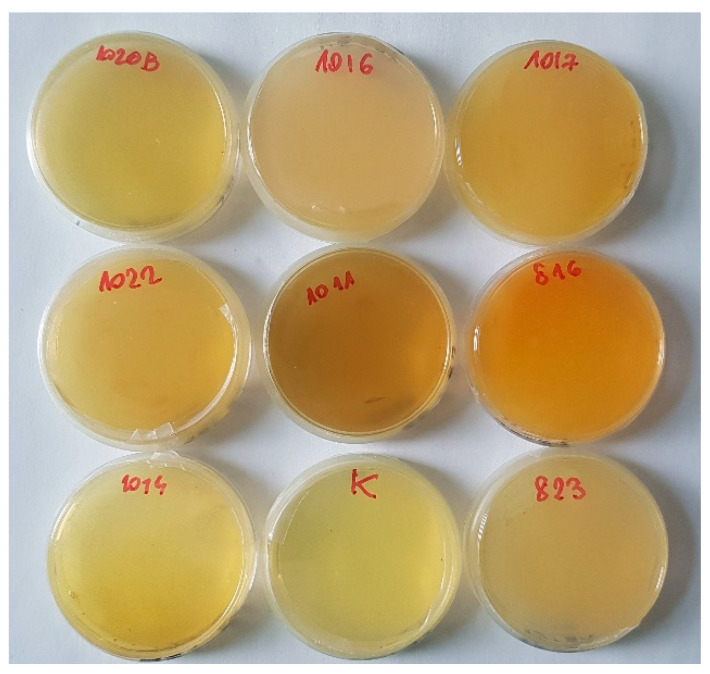
The cyanogenic activity of selected bacterial strains: the highest activity in *Serratia liquefaciens* (816); medium activity *Stenotrophomonas maltophila* (1020B), *Devosia insulae* (1014), *Tsukamurella tyrosinosolvens* (1017, 1011), low activity: *Aminobacter aminovorans* (1022), and *Microbacterium* sp. (1016).

In the environment, microorganisms utilize a wide spectrum of strategies to compete for and obtain resources. One of them is the production of organic and inorganic molecules, toxic for other organisms. One of these molecules is hydrogen cyanide (HCN) [24]. To verify whether bacteria associated with the fungus can produce hydrogen cyanide that could serve as a toxin to rotifers, we measured HCN production by selected bacterial strains associated with *Z. insidians*. Based on bacteria cultivation on LB (lysogeny broth) medium with glycine, which is a natural precursor of the hydrogen cyanide, the highest production was shown in *Serratia liquefaciens* (Figure 10). Others showed either no activities or had low activity.

## 3. Discussion

The investigations presented in this report describe the possible, complicated interplay between microorganisms inhabiting activated sludge. We found several bacteria involved in loose associations with the predatory fungus *Zoophagus intraradices,* and bacteriophages inhabiting these bacteria. One of these phages was identified as novel. Our results describe the possible relationships between organisms and show that they may be involved in a complicated trophic network that additionally involves *Lecane inermis,* a rotifer species that *Z. intraradices* preys on. According to our observations, bacteria enter in and out of cultured *Z. insidians*. This conclusion is based on the visualization of bacteria inside and outside the fungus and visualization of the bacterial entrance/exit process. Unfortunately, it is unknown which species are involved in this phenomenon. It is impossible to grow the fungus under aseptic culture, which could give us a chance to better understand the interaction, especially utilizing the FISH (fluorescence in situ hybridization) method to discriminate between individual bacterial strains. Bacteria migrating from in and out of the fungal hyphae were shown by Moebius et al. [25], who investigated the entrance of bacteria into the hyphae of *Rhizopus microsporus.* The only difference between the two models is that, in the case of *Z. insidians*, we did not observe the bacteria melting on the surface of the mycelial hyphae. In the case of *R. microsporus*, isolated bacteria were shown to employ extracellular enzymes as chitinase and other proteins to colonize living fungal hyphae. The *Bacillus* strain isolated in this study could also synthesize and secrete chitinase, suggesting its involvement in the active entrance into the fungal mycelium and possible digestion of the rotifer cuticle. Chitin is also present in the rotifer cuticle [26].

Due to the engulfment of bacteria, *R. microsporus* developed the ability to produce toxins that allow the fungus to change lifestyle from saprobic to pathogenic. The intensive green fluorescence of *Z. insidians* traps after staining with a Live–Dead Bacterial Kit suggests the presence of nucleic acid; however, we also could not distinguish whether it belonged to the fungus or bacteria. Previously, this material was described as an electron-dense substance released by the fungus [27], but our observations indicate that the substance may be of bacterial origin. As the traps’ tips were uniformly green, we suggest that this substance is either assemblages of unknown bacteria or the DNA produced at the top of the traps, subsequently released. Fungi and bacteria often produce so-called eDNA [28,29,30,31], which has strong gluing activity; however, this requires further studies. 

The staining of bacterial and fungal nuclei inside the mycelium gave us the possibility to discriminate them. Furthermore, they differed in size (nuclei were two-fold bigger), while both were stained by LIVE/DEAD markers as green (alive) or red (dead). The nuclei did not stain with oil red O while bacteria became red to dark brown (both outside and inside the hyphae) (as described by Shooter [32], oil O stains bacterial cell membranes). Unfortunately, we did not cure the fungi of bacteria, and the confirmation that bacteria can enter the mycelium has to wait for further studies. We could only make a comparative staining with both stainings on *Rhizopus* sp. and this confirmed our results. 

According to our observations, the rotifers that make contact with fungal hyphae are immobilized and intoxicated. Such a strategy is often used by predatory fungi and in other fungi (e.g., endophytic fungi that are growing within plants and can protect themselves from herbivores or saprobic fungi producing mycotoxins that keep competitors away), as reviewed by [33]. Our observation indicates that the intoxication of immobilized rotifers may result from the cooperative action of the fungus and its bacterial symbionts. The trap had a thinner wall at the tip (like all the growing hyphae tips), which could facilitate bacteria exiting from the trap. We can only speculate that bacteria can be released from the traps while the prey is caught. We could not distinguish whether the bacteria were leaving the traps or if only a glue-like substance was secreted to immobilize the rotifer. These are still only hypotheses, and further work is needed to understand the relationships between interacting organisms. 

*Zoophagus* belongs to Zoopagomycotina, the sister group of Mucoromycotina, (both previously included in Zygomycota), which are considered poor in secondary metabolites (except for carotenoids) [34]; thus, it seemed probable that the hypothetical toxin is produced as gene products of the phage (which was seen on the surface of bacteria) or bacteria. This would suggest that the symbiosis with bacteria (known for toxin production) could enrich the fungus in compounds that facilitate nutrient acquisition from rotifers. Additionally, bacterial chitinase production was shown on several occasions. According to the literature, chitinase may be responsible for the intoxication of various insects and other small animals [35]. We hypothesized that the enzyme might be involved in intoxicating or even the digestion of rotifers; however, we cannot rule out that it plays a different function such as the facilitation of the entrance of bacteria into the mycelium protected by a chitin cell wall and the release of bacteria from the mycelium. 

To verify whether the rotifers were sensitive to the enzyme, we treated rotifer cultures with commercially available bacterial chitinase. The effect of chitinase at the beginning was negligible as treatment with chitinase suspension buffer immobilized all rotifers; however, significant changes in rotifer mobility were apparent 24 h after treatment. We still do not know if the concentration of the enzyme used was representative of the rotifer–bacteria model investigated here. The concentration of chitinase produced at the tip of the trap is unknown; however, bacteria such as *S. maltophilia* can produce much higher chitinase concentrations in the medium [36]. Chitinase concentrations used in the present study were previously used in animal studies [37]. We should also emphasize that a negative result in the agar method should not be treated as ultimately eliminating the activity of this enzyme, as commonly, the phenotype of an organism depends on the environmental factors. As shown in agar tests, *Bacillus* sp. showed no chitinase production; however, chitinases from different organisms exhibit different specificity toward various substrates (chitin derivates); thus, we evaluated the chitinolytic activity of *Bacillus* sp. using three other substrates. The highest activity of the enzyme was detected in the sample supplemented with 4-Methylumbelliferyl N-acetyl-β-D-glucosaminide. The enzyme from *Bacillus* sp. was shown to belong to the group of exochitinases and is probably only active during the lysogenic stage of the *Bacillus* virus. Our previous demonstrations additionally supported the idea that various bacteriophages (viruses infecting bacteria) encode toxins [19]. Bacteria can use such toxins to kill unicellular eukaryotic predators including some ciliates such as *Tetrahymena* sp. [20,23]. Upon prophage induction by antibiotics, the phage switches from lysogenic to the lytic mode, and progeny virions are produced, and the bacterial cell is lysed. Such stress conditions causing prophage induction can arise from the attack of predators, which excrete hydrogen peroxide to combat bacteria [19].

Toxins are often produced due to the activities of their genes located in phage genomes. These genes are silent, but are activated upon prophage induction. If the efficiency of prophage induction is low, most cells in the bacterial population survive at the level of a few percent; however, the production of toxins by cells in which prophages were induced and their subsequent release following cell lysis may allow for the killing of the predator. A small fraction of bacteria that die due to prophage induction and cell lysis save the rest of the population as phage-encoded toxins eliminate the predator. Such a phenomenon has been described for Shiga toxin-producing *Escherichia coli*, which carry Shiga toxin-converting bacteriophages, and the phenomenon has been called ‘bacterial altruism’ [20]. Here, we asked if a similar process may allow the production of toxins in bacterial cells, which upon cell lysis, could facilitate the killing of rotifers trapped by the predatory fungus. To test if isolates of bacteria are lysogenic for bacteriophages, we provoked the phage lytic development by adding mitomycin C to the bacterial cultures. Another option is that rotifers could be toxified by hydrogen cyanide produced by bacteria and released into the environment. Indeed, some bacteria isolated from *Zoophagus* can produce cyanide, as shown in our experiment with the agar test.

Interestingly, we observed phages leaving the bacterial cells using a transmission electron microscope, suggesting that the tested bacteria contained prophage DNA integrated into the chromosome. The identified virus from *Bacillus* sp. has not been previously described, but its discovery did not bring us closer to understanding the involvement of viruses in rotifer immobilization. Undoubtedly, other lytic enzymes can be important in the digestion of rotifers, and non-enzymatic mechanisms could be involved [38,39]. According to these reports, the production of lytic enzymes enables the bacterium to outcompete fungi and G+ bacteria for habitat and resources.

## 4. Materials and Methods

### 4.1. Isolation of Predatory Fungi 

The predatory fungus strain was isolated from the activated sludge samples from a wastewater treatment plant located in the south of Poland in 2015. The samples were isolated from nitrification chambers and transported to the laboratory within 24 h. One milliliter subsamples of thoroughly mixed activated sludge were transferred to the wells of tissue test plates (Cell Wells™, TPP) and *Lecane inermis* rotifers were provided as a food source. When the mycelium grew on the bottom, the sludge was delicately removed with a pipette and Żywiec brand spring water was added to the wells along with a fresh portion of rotifers. The procedure was repeated several times and the cultures were incubated at a temperature of 20–21 °C in darkness. To obtain the strains, appearing conidia were transferred individually to separate wells filled with Żywiec water, and again, the rotifers were added. Then, the best growing strain was chosen for further cultivation. 

### 4.2. Cultivation of Predatory Fungi and Rotifers and Isolation of Endomycelial Bacteria 

*Zoophagus insidians* were grown in commercial mineral water (Żywiec, Poland) in multi-well plates. The culture was fed with rotifers for ten days. After this period, feeding was stopped to starve the fungus. To identify endophytic bacteria (without epiphytes/microorganisms inhabiting its surface) from the fungus, the mycelium was subject to two different treatments. (1) The mycelium was suspended in 1 mL sterile 0.9% NaCl solution, transferred to a 1.5 mL Eppendorf tube centrifuged at 1500× *g* for 1 min. The mycelium was then rinsed with sterile water 25 times, crushed with a micropestle, and suspended in 1 mL sterile 0.9% NaCl solution. The water from the last washing was used as a negative control of the surface sterilization process. Subsequently, 90 μL of the suspension was withdrawn and plated on solid media. The following nutrient media were selected for bacterial isolation: (i) PYG [40]; (ii) N-free medium [41]; and (iii) 337a [42]. Additionally, 300 μL of the suspension was provided for direct molecular identification of the fungus and endohyphal bacteria species (polymerase chain reaction, DNA cloning, and sequencing). (2) Water from the wells was removed using a pipette and the mycelium was suspended in 3 mL of “Żywiec” sterile water supplemented with the following antibiotics: kanamycin (60 μg mL^−1^), streptomycin (60 μg mL^−1^), chloramphenicol (60 μg mL^−1^), and ciprofloxacin (100 μg mL^−1^). Antibiotics were dissolved in water except for chloramphenicol (96% ethanol) and added using a sterile syringe filter (PTFE (Polytetrafluoroethylene), 0.22 μm). The plates were shaken for 1 min (60 rpm) and placed in the dark at 21 °C for 24 h. Antibiotic treatment was repeated three times. Samples from every second well were then transferred to a Falcon tube and spun for 2 min at 2000 rpm min^−1^ (from six wells, three samples were obtained). After centrifugation, the supernatant was removed and the mycelium was rinsed by adding sterile water and centrifuged for 2 min at 2000× *g*. Rinsing was repeated three times. Water from the last rinsing was used as control of the sterilization process. The mycelium was crushed with a “micropestle” and suspended in 1.5 mL of sterile commercial water (Żywiec). Then, 90 μL of the suspension was withdrawn and plated on solid nutrient media for bacterial isolation, as above-mentioned. Additionally, a modified Schleger medium [43] was used. The Petri-dishes were placed in the dark (three plates × four medium × three samples = 36 plates, + four plates with water) at 28 °C for a period of 14 days. Then, 300 μL of the suspension was collected for DNA isolation to verify the fungal species.

### 4.3. Identification of the Fungus and Bacteria 

According to morphological features, the fungus was classified as *Zoophagus* sp. [22]. To verify the identification, a molecular approach was employed. DNA extraction from the mycelium was performed according to Azmat et al. [44]. NucleoSpin^®^ gDNA Clean-up (Macherey-Nagel, Düren, Germany) was used to purify the DNA. Amplification of the 18S rDNA region was carried out with Pf1fw (5′-CAAGTCTGGTGCCAGCAGC-3′) and Pf2rev (5′-GACTACGACGGTATCTGATC-3′) primers [45]. For the PCR, 10 µg of DNA template, 10 pmol of each primer, and Maxima Hot Start Green PCR Master Mix (Thermo Fisher Scientific, Waltham, MA, USA) were used. PCR was performed in the following conditions: (1) an initial denaturation at 95 °C for 4 min; (2) 30 cycles consisting of denaturation at 95 °C for 30 s, annealing at 55 °C for 30 s, and elongation at 72 °C for 45 s; and (3) a final elongation at 72 °C for 5 min. PCR products were visualized in 1.5% agarose gel (Prona, Germany) stained with SimplySafe (Eurx, Poland). The StrataClone PCR cloning system was used for the cloning of PCR products (Stratagene, Agilent, USA). Briefly, Taq-amplified PCR products were ligated into the vector. The linear molecule was then transformed into a competent cell line expressing Cre recombinase. Circulated PCR products were replicated in cells growing on LB-based agar medium supplemented with Ampicillin, XGal, and IPTG (Isopropyl β-d-1-thiogalactopy). Ten colonies harboring the plasmid containing PCR product inserts after overnight incubation at 37 °C were selected to amplify the 18S rDNA, performed according to the description above. Since the PCR products were not visible in electrophoresis, nested PCR with the same primers at the same conditions was carried out. The PCR products were sequenced by Macrogen (The Netherlands). The Pf1fw and Pf2rev primers were used for reading the sequences. Nucleotide sequences were analyzed with Chromas (www.technelysium.com.au) and Bioedit software and compared with the published sequences in the NCBI database (www.ncbi.nlm.nih.gov) using the BLASTN algorithm. For species identification, a threshold of 98% sequence similarity to the reference sequence was applied. 

Three different modes of endohyphal bacteria isolation were applied: (1) direct amplification DNA from water-sterilized mycelium; (2) amplification of DNA of pure bacterial cultures isolated from water-sterilized mycelium; and (3) amplification of DNA of pure bacterial cultures isolated from antibiotic-sterilized mycelium.

DNA extraction from bacteria was carried out by mini kit DNA reagents (Syngen, Poland). The concentration and purity of DNA were determined spectrophotometrically. The 16S rDNA fragment was then amplified using the 27F (5′-GAGTTTGATC CTGGCTCAG-3′) and 1492R (5′-GGTTACCTTGTTACGACTT-3′) primers [46] and the Maxima Hot Start Green PCR Master Mix reagents (Thermo Fisher Scientific, USA). PCR was performed according to the details described above, with the strand elongation step extended to 90 s. The size of the PCR products was verified by agarose gel electrophoresis and sequenced by Macrogen (Amsterdam, The Netherlands) using 27F and 1492R primers. The nucleotide sequences were analyzed as described for the fungus. In the case of unsuccessful identification of bacteria to the species level, rpoB (beta subunit RNA polymerase) sequence with primers rpoB1698F (5′-AACATCGGTTTGATCAAC-3′) and rpoB2041R (5′-CGTTGCATGTTGGTACCCAT-3′) and 16S-ITS-23S rDNA fragment with primers 16S-870F (5′-CCTGGGGAGTACGGTCGCAAG-3′) and FGPL54′ (5′-CCGGGTTTCCCCATTCGG-3′) were amplified, sequenced, and analyzed [47,48]. For the first mode of sample preparation (direct amplification DNA from the mycelium), the StrataClone PCR cloning system was used for the cloning of PCR products (Stratagene, Agilent, Santa Clara, California, USA). Besides using a variety of primer pairs, *Bacillus* sp. was not identified to the species level.

### 4.4. Localization and Viability of Bacteria and Fungi 

To verify the viability of the fungus and co-occurring organisms, cultures (microorganisms suspended in sterile water) were established on 10 × 10 mm cover glasses in 5 cm Petri dishes (instead of multi wells). The LIVE/DEADTM Yeast Viability Kit and LIVE/DEAD BacLight TMBacterial Viability Kit (Invitrogen detection technologies) were used to visualize the microorganisms present in the culture according to the manufacturer’s protocol. Samples were stained on cover glasses with PBS buffer (0.5 mL) containing 2 µL of each staining solution: A and B, at room temperature in the darkness for ca. 15 min, followed by gentle washing with PBS, mounted on the glass slide. Samples were examined using a NIKON Eclipse fluorescent microscope and confocal microscope. Additionally, the material adhered to cover glasses was stained with calcofluor white and oil red O to visualize lipid droplets [49].

### 4.5. Scanning Electron Microscopy 

The fungi were cultivated on 9 mm, circular cover glasses (Agar Scientific Ltd., Stansted, UK) in Petri dishes (as described above) for a week. After this time, cultures were gently washed with a fresh culture medium and fixed for 30 min with a mixture of 4% formaldehyde (158127, Sigma-Aldrich, St. Louis, MO, USA) and 2% glutaraldehyde (01909, Polysciences, Inc., Warrington, FL, USA) in 0.1 M cacodylic buffer (15540.02, Serva Electrophoresis GmbH, Heidelberg, Germany). Next, the specimens were washed three times with a 0.1 M cacodylic buffer and dehydrated with increasing ethanol concentrations (POCH, Gliwice, Poland). Finally, they were transferred to 1:1 (*v:v*) mixture of ethanol:acetone followed by incubation in dehydrated acetone (POCH, Gliwice, Poland) and drying at a critical point (CPD E3000, Quorum Technologies, Lewes, UK). The dried samples (still on cover glasses) were adhered to microscope holders and sputter coated with ~10 nm layer of gold (JFC-1100E, JEOL, Tokyo, Japan). The specimens were imaged using a JEOL JSM5410 scanning electron microscope (JEOL, Tokyo, Japan). Similar samples coated with carbon (JEC-530, JEOL, Tokyo, Japan) were observed using Hitachi S-4700 FEG scanning (Institute of Geology of the Jagiellonian University in Kraków, Poland).

### 4.6. Transmission Electron Microscopy

The fungal mycelium was transferred to Eppendorf tubes containing 1 mL of fixative (a mixture of 4% formaldehyde and 2% glutaraldehyde in 0.1 M cacodylic buffer). The specimens were fixed overnight at 4 °C and washed three times in 0.1 M cacodylic buffer followed by 1 h incubation in a 1% OsO_4_ (0223B, Polysciences Inc., Warrington, PA, USA) water solution. After contrast, the samples were dehydrated using increasing ethanol concentrations and transferred to propylene oxide (00236-1, Polysciences, Inc., USA). After 20 min, the samples were infiltrated overnight with a propylene oxide:resin (Epon Resin 828, 02334, Polysciences, Inc., USA) mixture (1:1) and incubated for 3 h in 100% resin. Subsequently, the resin was exchanged, and the samples were incubated overnight at 37 °C. The sections (60 nm) were cut using an ultramicrotome (Leica EM UC7, Austria), and the images were collected using a JEOL JEM2100 HT transmission electron microscope (JEOL, Tokyo Japan).

### 4.7. Prophage Induction

To test whether isolates of bacteria are lysogenic with bacteriophages, host cells were cultured in liquid tryptic soy broth (TSB) with aeration at 30 °C in a shaking incubator (200 rpm; Eppendorf). All tested bacteria are listed in Table S1. Cultures were diluted to OD600 = 0.2. The phage lytic development was provoked by the addition of mitomycin C to a final concentration of 1 µg mL^−1^. In control experiments, water (a solvent used to prepare stock solutions) was added instead of the antibiotic. The cultivation was continued at 30°C for 20 h. The presence of bacterial cell lysis suggested that the host cells contain phage DNA integrated into the chromosome. To confirm the obtained results, the prophage induction procedure was performed according to the double overlay plaque assay with minor modifications [50,51]. To obtain visible plaques formed by bacteriophages, Petri dishes were filled with 25 mL of tryptic soy agar (TSA) supplemented with 5% glycerol and a sublethal concentration of the appropriate antibiotic: ampicillin (3.5 µg µg mL^−1^), chloramphenicol (2.5 µg mL^−1^), or tetracycline (1.5 µg mL^−1^). In the next step, 2 mL of the top TSB agar (contain 0.4% agarose) was mixed with 1 mL of the overnight bacterial cell culture and the prophage inductor mitomycin C to a final concentration of 1 µg mL^−1^ (Table S1). The mixture was poured onto the previously prepared bottom agar. The plates were dried 10 min at room temperature and then incubated at 30 °C for 20 h. The process of prophage induction was confirmed by the presence of clear zones on the bacterial lawn. The areas of the bacterial lawn that looked no different from the control samples (not treated with a prophage inductor) were scored as negative.

### 4.8. The Effect of Phage Lysate and Chitinase on the Viability of Rotifers

Endohyphal bacterial strains: *Chrysobacterium daeguense* (strain 824), *Ochrobactrum thiopenivorans* (strain 1015), *Pseudomonas alcaligenes* (strain 813), *Achromobacter xylosoxidans* (strain 826), *Serratia liquefaciens* (strain 816), *Devosia insulae* (strain 1014), *Tsukamurella tyrosinosolvens* (strains 1011 and 1017), *Paenibacillus cineris* (strain 1001), *Microbacterium* sp. (strains 1007 and 1016), *Bacillus* sp. (strain 1020A), and *Stenotrophomonas maltophilia* (strain 1020B) were tested for their effect on rotifer activity. Bacteria were cultured in TSB medium at 30 °C to OD600 = 0.2. The culture was divided into two aliquots. One was treated with mitomycin C (1 µg mL^−1^) to provoke prophage induction. The second was a control sample without an induction agent. After overnight cultivation at 30°C, bacterial samples were harvested by centrifugation (4000× *g*, 20 min, 4°C). The supernatants were collected and filtered through PVDF (polyvinylidene difluoride) membrane filters with a pore size of 0.22 µm to remove bacterial cells. The same procedure was used for the preparation of additional control samples that contained TSB medium or TSB medium with mitomycin C. In the next step, the influence of filtered samples on the viability of rotifers was tested. Rotifers were cultured in a tissue culture plate (24 wells each 250 µL) and the ratio of motile to immobilized rotifers at five randomly selected sites in each well was measured. Subsequently, 10 µL of phage culture was added to wells with rotifers (n = 3), shaken for 5 s, and kept in darkness at 20 °C. After the following 2 h, the ratio of motile to immobilized rotifers was measured. The following controls were used in the experiment: (a) bacteria pure culture in TSB medium; (b) TSB medium; and (c) TSB medium supplemented with mitomycin C. Rotifer motility index was calculated as a ratio of percent of motile rotifers treated with phages to the percent of motile rotifers treated with TSB medium supplemented with mitomycin. 

The same system was used to study the influence of commercially available chitinase (Sigma-Aldrich, USA) dissolved in PBS (phosphate buffer saline) on rotifers. Final chitinase concentrations used in the experiment were 0.0001, 0.001, and 0.01 U. The production of chitinase by the bacterial strains was tested on an agar medium supplemented with colloidal chitin from crab shells (Sigma-Aldrich, St. Louis, MO, USA). The strains were compared by measuring the halo around the colonies. Colloidal chitin was prepared with the method described in [52]. Briefly, 5 g of chitin was added to 60 mL HCl, stirred at room temperature for 1 h, and filtered through glass wool. The filtrate was stirred with 200 mL of 50% ethanol. The precipitate was washed with sterile water until colloidal chitin reached pH = 7.0.

### 4.9. Extraction of Bacteriophage DNA from Bacillus sp. 1020A Lysate

The liquid culture of *Bacillus* sp._1020A was grown with aeration at 30 °C to OD600 = 0.2. Induction of prophages was provoked by the addition of mitomycin C to a final concentration of 1 µg mL^−1^. The following day, the obtained phage lysate was clarified by centrifugation (4000× *g*, 20 min, 4°C) and the genomic DNA of *Bacillus* sp. 1020A phage was isolated with the Phage DNA Isolation Kit of Norgen Biotek Corporation (Ontario, Canada), according to the manufacturer’s instructions. The concentration and purity of the extracted genomic DNA were confirmed by spectrophotometric and agarose gel analyses.

### 4.10. Sequencing and Bioinformatic Analysis of Phage vB_Bacillus_1020 Genome

The vB_*Bacillus*_1020 bacteriophage genome was sequenced by using an Illumina HiSeq genome sequencer at the Eurofins Genomics company (Luxembourg, Grand Duchy of Luxembourg). The raw data (5,859,980 raw reads) were processed to obtain high-quality clean reads using Trimmomatic to remove adapter sequences, ambiguous reads, and low-quality sequences. The processed reads were de novo assembled using the CLC assembler (CLC Genomics Workbench). Putative open reading frames (ORFs) were predicted by RASTtk software and verified by BLAST and InterProScan 5 analysis [53,54]. The putative function of translated products was annotated by BLASTp and PHASTER Prophage/Virus databases [55]. The NCBI’s Conserved Domain Database was also used for predicting protein domains and motifs. ShortBRED was used to search the virulence factors and toxins in predicted ORFs against the Virulence Factors of Pathogenic Bacteria database (VFDB) [56,57]. To generate the circular map of Bacillus sp. 1020A bacteriophage genome, perform GC skew, and GC content analyses, CGView was employed. The genome sequence of the vB_Bacillus_1020 bacteriophage genome was deposited in GenBank under the accession number: MT210152.

### 4.11. Determination of Bacteriophage vB_Bacillus_1020 Host Range

The host range of phage vB_Bacillus_1020 was determined by a standard spot test, using a phage lysate of the titer of 4.3·10^10^ phages·mL^−1^ with the following bacterial strains: *B. subtilis* 168 (Collection of the Department of Molecular Biology of University of Gdansk, Poland), *B. subtilis* 3610 (Collection of the Department of Molecular Biology of University of Gdansk), *B. subtilis* KPD 1329 (Collection of Plasmids and Microorganisms of University of Gdansk), *B. subtilis* KPD 1330 (Collection of Plasmids and Microorganisms of University of Gdansk), *B. cereus* KPD 1331 (Collection of Plasmids and Microorganisms of University of Gdansk), *B. megaterium* KPD 1332 (Collection of Plasmids and Microorganisms of University of Gdansk), *B. firmus* DSM 12 (DSMZ—German Collection of Microorganisms and Cell Cultures GmbH, Braunschweig, Germany), and *B. siralis* DSM 13140 (DSMZ—German Collection of Microorganisms and Cell Cultures GmbH, Braunschweig, Germany). Briefly, 1 mL of overnight bacterial culture was mixed with 2 mL of soft agar poured onto plates filled with bottom agar. The phage lysate of vB_Bacillus_1020 was diluted in TM buffer (10 mM Tris-HCl, 10 mM MgSO_4_; pH 7.2), and 10 μL of each dilution was spotted onto the top agar. After a 24 h incubation at 37°C, bacterial sensitivity to vB_Bacillus_1020 infection was analyzed.

### 4.12. Cyanogenic Activity of Bacterial Strains

To verify the ability of isolated bacterial strains to produce hydrogen cyanide, HCN (hydrogen cyanide) production was analyzed using a standard functional screening method [58]. Each bacterial strain was tested in a separate Petri dish. LB medium was supplemented with glycine (0.5 g L^−1^) and autoclaved. Filter papers were autoclaved, saturated with 800 µL of 1.3% picric acid (Sigma), left overnight, treated with a few drops of 10% sodium carbonate, and stuck to the upper part of the Petri dishes, over the bacterial cultures pre-grown 24 h before. Negative control was without bacteria. The cultures were checked every 24 h. The change of color from yellow to orange/red indicated the production of hydrogen cyanide.

### 4.13. Statistical Methods

Statistical analyses were carried out with Statistica software ver. 13 (TIBCO) at the level of significance *p* ≤ 0.05. Data normality and variance homogeneity were evaluated by the Shapiro–Wilk and Levene’s tests, respectively. Statistical significance was determined by analysis of variance (ANOVA), followed by Tukey’s *post-hoc* test. For non-parametric data, the Kruskal–Wallis test was used.

## 5. Conclusions

Can any lessons regarding activated sludge or other water bodies be drawn from this research? The development of symbiosis seems to be an adaptive trait. Indeed, the fungus either shares nutrients with bacteria or bacteria release nutrients taken up by the fungus. This may be why the predatory fungi in water bodies succeed, although this phenomenon is mostly overlooked. Bacteriophages abundantly present on the surface of bacteria that migrate from inside of the fungal traps are another interesting issue requiring further investigation. Understanding the relationship between predatory fungi associated bacteria and bacteriophages and the mechanism of their interaction with their prey is essential due to the increasing number of activated sludge cleaning plants experiencing disorders associated with predatory activity fungi. If we can solve this mystery, perhaps it would be possible to control activated sludge functioning by increasing the wastewater treatment plants with observed effects of predatory fungi activity. On the other hand, it is worth noting that the slow decomposition of rotifers is often a problem in water reservoir trophic networks and particulate organic matter (POM) transport. The predatory fungi might also be useful. Despite much greater bacterial activity inside carcasses of rotifers than outside their bodies, the degradation of rotifers is prolonged, caused by the presence of proteins resistant to biodegradation [59]. Another reason why rotifers are degraded slowly is the body shell, which is made of keratin (resistant to degradation) and chitin [26]. In the case of POM accumulating in rotifer carcasses [59], predatory fungi may be helpful. The phenomena described above indicate the acceleration of degradation of carcasses by the *Zoophagus*-related microorganisms. It should be mentioned here that other predatory fungi are commonly used against nematodes causing losses in agriculture. Potentially, this could also be the case of the described above symbiosis. The chitinase of bacteria is known to help in the development of new products useful in medicine, agriculture, and industrial applications including hypocholesterolemic and antihypertensive activity, food quality enhancers [60] as well as antifungal and antibacterial therapy, especially in cases when biofilm formation is undesirable [17,61,62]. Nevertheless, it seems to us that the role of these bacteria in the development of new evolutionary traits is also an essential element to understand evolution. In this light, deciphering complex mechanisms of interactions between the cells of microorganisms and bacteriophages, which form sophisticated trophic networks, appears particularly important and intriguing. Bacteriophages have been previously proposed to play important roles in the interactions between bacteria in which they occur as prophages and unicellular eukaryotic predators. However, in this report, we present a proposal that these viruses are involved in even more complicated interactions, forming a quadruple network between phages, bacteria, fungi, and rotifers. This indicates another level of complexity between natural microbial interactions.

## Figures and Tables

**Table 1:** Molecular identification of bacteria isolated from *Zoophagus insidians*.

Bacterial Strain No UNIJAG.PL.	Gene	NCBI Accession Number	Identification	Best Match Sequence/Accession Number	Similarity
826	16S rDNA	MT385270	*Achromobacter xylosoxidans*	*Achromobacter xylosoxidans* CP014060.1	1219/1220 (99%)
				*Achromobacter spanius* KX527629.1	1218/1220 (99%)
				*Achromobacter insolitus* KC633947.1	1218/1220 (99%)
811	16S rDNA	MT385259	*Acidovorax delafieldii*	*Acidovorax delafieldii* JQ689177.1	1130/1136 (99%)
812	16S rDNA	MT385260	*Acidovorax delafieldii*	*Acidovorax delafieldii* JQ689177.1	1001/1004 (99%)
814	16S rDNA	MT385262	*Acidovorax delafieldii*	*Acidovorax delafieldii* JQ689177.1	925/927 (99%)
815	16S rDNA	MT385263	*Acidovorax delafieldii*	*Acidovorax delafieldii* GQ284435.1	831/962 (86%)
824	16S rDNA	MT385268	*Chryseobacterium daeguense*	*Chryseobacterium daeguense* NR_044069.1	1018/1028 (99%)
813	16S rDNA	MT385261	*Pseudomonas alcaligenes*	*Pseudomonas alcaligenes* LT223677.1	1132/1135 (99%)
816	16S rDNA	MT385264	*Serratia liquefaciens*	*Serratia liquefaciens* MF083085.1	1278/1279 (99%)
823	16S rDNA	MT385267	*Serratia liquefaciens*	*Serratia liquefaciens* MF083085.1	1054/1054 (100%)
817	16S rDNA	MT385265	*Stenotrophomonas maltophilia*	*Stenotrophomonas maltophilia* JQ897943.1	598/604 (99%)
822	16S rDNA	MT385266	*Stenotrophomonas maltophilia*	*Stenotrophomonas maltophilia* KU726005.1	1153/1153 (100%)
				*Stenotrophomonas acidaminiphila* KR822265.1	1153/1153 (100%)
				*Stenotrophomonas humi* KR822264.1	1153/1153 (100%)
825	16S rDNA	MT385269	*Stenotrophomonas maltophilia*	*Stenotrophomonas maltophilia* KU726005.1	1209/1211 (99%)
				*Stenotrophomonas acidaminiphila* KR822265.1	1209/1211 (99%)
				*Stenotrophomonas humi* KR822264.1	1209/1211 (99%)
827	16S rDNA	MT385271	*Stenotrophomonas maltophilia*	*Stenotrophomonas maltophilia* KU726005.1	1220/1220 (100%)
				*Stenotrophomonas acidaminiphila* KR822265.1	1220/1220 (100%)
				*Stenotrophomonas humi* KR822264.1	1220/1220 (100%)
1020A	16S rDNA	MT385285	*Bacillus* sp.	*Bacillus siralis* KP282785.1	1099/1100 (99%)
				*Bacillus firmus* HM030743.1	1099/1101 (99%)
1020B	16S rDNA	MT385286	*Stenotrophomonas maltophilia*	*Stenotrophomonas maltophilia* CP040735.1	1361/1363 (99%)
1024	16S rDNA	MT385288	*Achromobacter xylosoxidans*	*Achromobacter xylosoxidans* CP014060.2	1190/1191 (99%)
				*Achromobacter insuavis* MF033478.1	1189/1191 (99%)
				*Achromobacter spanius* KX527629.1	1189/1191 (99%)
	rpoB	MT436219		*Achromobacter xylosoxidans* CP014060.2	275/278 (99%)
	16S-23S rDNA	MT431664		*Achromobacter xylosoxidans* CP014060.2	1009/1025 (99%)
1012	16S rDNA	MT385279	*Aminobacter aminovorans*	*Aminobacter aminovorans* KT597534.1	1171/1171 (100%)
				*Carbophilus carboxidus* MF077161.1	1155/1155 (100%)
	rpoB	MT436217		*Aminobacter aminovorans* CP015005.1	281/297 (95%)
1013	16S rDNA	MT385280	*Aminobacter aminovorans*	*Aminobacter aminovorans* MF093200.1	1090/1091 (99%)
1022	16S rDNA	MT385287	*Aminobacter aminovorans*	*Aminobacter aminovorans* MF093200.1	1202/1202 (100%)
				*Carbophilus carboxidus* MF077162.1	1201/1201 (100%)
	rpoB	MT436218		*Aminobacter aminovorans* CP015005.1	282/298 (95%)
1023	16S rDNA		*Bacillus* sp.	*Bacillus circulans* MG020100.1	864/1071 (81%)
1006	16S rDNA	MT385275	*Bosea vestrisii*	*Bosea vestrisii* MF101196.1	1098/1098 (100%)
1003	16S rDNA	MT385274	*Bosea/Starkeya*	*Bosea lupini* KF730777.1	1346/1351 (99%)
				*Bosea robiniae* NR_108516.1	1346/1351 (99%)
				*Afipia geno* sp. U87773.1	1346/1352 (99%)
				*Starkeya* sp. JX219400.1	1344/1351 (99%)
	16S-23S rDNA	MT431661		*Starkeya* sp. JX219400.1	473/523 (90%)
				*Bosea* sp. CP022372.1	457/525 (87%)
1009	16S rDNA	MT385277	*Bosea/Starkeya*	*Bosea lupini* KF730777.1	1167/1170 (99%)
				*Bosea robiniae* NR_108516.1	1167/1170 (99%)
				*Afipia geno* sp. U87773.1	1169/1170 (99%)
				*Starkeya* sp. JX219400.1	1167/1170 (99%)
	16S-23S rDNA	MT431662		*Starkeya* sp. JX219400.1	977/1007 (97%)
				*Bosea* sp. CP022372.1	965/1009 (96%)
1014	16S rDNA	MT385281	*Devosia insulae*	*Devosia insulae* NR_044036.1	1081/1083 (99%)
1007	16S rDNA	MT385276	*Microbacterium* sp.	*Microbacterium oxydans* MH211289.1	1138/1138 (100%)
				*Microbacterium phyllosphaerae* KY936459.1	1138/1138 (100%)
				*Microbacterium foliorum* CP019892.1	1138/1138 (100%)
1016	16S rDNA	MT385283	*Microbacterium* sp.	*Microbacterium oxydans* MH211289.1	1169/1169 (100%)
				*Microbacterium phyllosphaerae* MF541529.1	1169/1169 (100%)
				*Microbacterium foliorum* MF681940.1	1169/1169 (100%)
1001	16S rDNA	MT385272	*Paenibacillus cineris*	*Paenibacillus cineris* KT831432.1	1199/1199 (100%)
1002	16S rDNA	MT385273	*Paenibacillus pabuli*	*Paenibacillus pabuli* NR_113627.1	1130/1130 (100%)
				*Paenibacillus taichungensis* NR_004428.1	1130/1130 (100%)
	rpoB	MT436216		*Paenibacillus pabuli* AY728291.1	319/331 (96%)
1015	16S rDNA	MT385282	*Ochrobactrum thiophenivorans*	*Ochrobactrum thiophenivorans* KY819002.1	1136/1136 (100%)
1011	16S rDNA	MT385278	*Tsukamurella tyrosinosolvens*	*Tsukamurella tyrosinosolvens* MG763891.1	1075/1075 (100%)
1017	16S rDNA	MT385284	*Tsukamurella tyrosinosolvens*	*Tsukamurella tyrosinosolvens* MH393216.1	1137/1137 (100%)
1021	16S-23S rDNA	MT431663	*Tsukamurella tyrosinosolvens*	*Tsukamurella tyrosinosolvens* CP019066.1	841/854 (98%)

**Table 2:** Chitinase activity of bacterial strains.

Bacterial Strain	Chitinase Activity (*)		
	No Activity	Low	High
*Tsukamurella tyrosinosolvens* UNIJAG.PL.1011			*
*Tsukamurella tyrosinosolvens* UNIJAG.PL.1017			*
*Tsukamurella tyrosinosolvens* UNIJAG.PL.1021			*
*Achromobacter xylosoxidans* UNIJAG.PL.826	*		
*Stenotrophomonas maltophilia* UNIJAG.PL.817		*	
*Stenotrophomonas maltophilia* UNIJAG.PL.822		*	
*Stenotrophomonas maltophilia* UNIJAG.PL.825			*
*Stenotrophomonas maltophilia* UNIJAG.PL.827			*
*Stenotrophomonas maltophilia* UNIJAG.PL.1024B			*
*Devosia insulae* UNIJAG.PL.1014	*		
*Aminobacter aminovorans* UNIJAG.PL.1012			
*Aminobacter aminovorans* UNIJAG.PL.1013			*
*Aminobacter aminovorans* UNIJAG.PL.1022			*
*Bacillus* sp. UNIJAG.PL.1023	*		
*Ochrobactrum thiophenivorans* UNIJAG.PL.1015		*	
*Microbacterium* sp. UNIJAG.PL.1007		*	
*Microbacterium* sp. UNIJAG.PL.1016			*
*Serratia liquefaciens* UNIJAG.PL.816	*		
*Serratia liquefaciens* UNIJAG.PL.823	*		
*Acidovorax delafieldii* UNIJAG.PL.811		*	
*Acidovorax delafieldii* UNIJAG.PL.812		*	
*Acidovorax delafieldii* UNIJAG.PL.814		*	
*Acidovorax delafieldii* UNIJAG.PL.815		*	
*Pseudomonas alcaligenes* UNIJAG.PL.813		*	
*Chryseobacterium daeguense* UNIJAG.PL.824	*		
*Paenibacillus cineris* UNIJAG.PL.1001			*
*Paenibacillus pabuli* UNIJAG.PL.1002			*
*Bosea/Starkeya* UNIJAG.PL.1003			*
*Bosea vestrisii* UNIJAG.PL.1006	*		
*Bosea/Starkeya* UNIJAG.PL.1009	*		

## Data Availability

The sequence of *Bacillus* sp. 1020A bacteriophage genome was deposited with GenBank under the accession number: MT210152.

## Supplementary Materials

The following are available online at https://www.mdpi.com/1422-0067/22/4/2178/s1.
