# Supplementary material for: Extraordinary Multi-Organismal Interactions Involving Bacteriophages, Bacteria, Fungi, and Rotifers: Quadruple Microbial Trophic Network in Water Droplets
Source: Int J Mol Sci. 2021 Feb 22;22(4):2178. doi: 10.3390/ijms22042178 (PMC7926626; doi:10.3390/ijms22042178)
Supplement: Supplementary file 1 [file ijms-22-02178-s001.pdf]

Table 1. The activation of prophage induction process with mitomycin C. Differences in bacterial growth relative to the control experiments were marked as (+). The (-) symbol represents the lack of changes between samples not- and treated with prophage inductor. Each experiment was repeated three times.

**Bacteria**	**Liquid TSB medium with mitomycin C (1 µg/ml)**	**TSA medium with 5% glycerol and sublethal concentration of appropriate antibiotic**		
		**Ampicillin (3.5 µg/ml)**	**Chloramph-enicol (2.5 µg/ml)**	**Tetracycline (1.5 µg/ml)**
Tsukamurella tyrosinosolvens DNA_1011	+	-	-	-
Tsukamurella tyrosinosolvens DNA_1017	+	+	+	+
Tsukamurella tyrosinosolvens DNA_1021	+	+	+	+
Mixture of Bacillus sp. DNA_1020 and *Stenotrophomonas malthopila*	+	+	+	+
Achromobacter xylosoxidans DNA_826	+	-	-	-
Achromobacter xylosoxidans DNA_1024	-	-	-	-
Stenotrophomonas maltophilia DNA_ 817	-	-	-	-
Stenotrophomonas maltophilia DNA_ 822	-	-	-	-
Stenotrophomonas maltophilia DNA_ 825	-	-	-	-
Stenotrophomonas maltophilia DNA_ 827	-	-	-	-
Devosia insulae DNA_1014	+	+	+	+
Aminobacter aminovorans DNA_1012	-	-	-	-
	-	-	-	-

Aminobacter aminovorans DNA_1013				
Aminobacter aminovorans DNA_1022	-	-	-	-
Microbacterium sp. DNA_1007	+	+	+	+
Microbacterium sp. DNA_1016	+	+	+	+
Serratia/Aranicola DNA_816	+	-	+	-
Serratia/Aranicola DNA_823	-	-	-	-
Acidovorax delafieldii DNA_811	-	-	-	-
Acidovorax delafieldii DNA_812	-	-	-	-
Acidovorax delafieldii DNA_814	-	-	-	-
Acidovorax delafieldii DNA_815	-	-	-	-
Pseudomonas alcaligenes DNA_813	+	+	-	-
Chryseobacterium daeguense DNA_824	+	-	+	-
Paenibacillus cineris DNA_1001	+	-	+	-
Paenibacillus pabuli DNA_1002	+	+	+	+
Bosea/Starkeya DNA_1003	-	-	-	-
Bosea vestrisii DNA_1006	-	+	+	+
Bosea/Starkeya DNA_1009	-	-	-	-
Ochrobactrum thiophenivorans DNA_1015	+	-	-	-
Bacillus sp. DNA_1023	-	-	-	-
